# Informing the design of embedded pragmatic trials of dementia care interventions: perspectives from a Lived Experience Panel

**DOI:** 10.1002/alz.086463

**Published:** 2025-01-09

**Authors:** Yaideliz M Romero‐Ramos, Carolyn Malone, Monica Moreno, Katherine Brandt, Gary Epstein‐Lubow

**Affiliations:** ^1^ Brown University School of Public Health, Providence, RI USA; ^2^ Alzheimer’s Association, Chicago, IL USA; ^3^ Massachusetts General Hospital, Boston, MA USA; ^4^ Frontotemporal Disorders Unit, Charlestown, MA USA; ^5^ Alpert Medical School of Brown University, Providence, RI USA

## Abstract

**Background:**

The National Institute on Aging (NIA) Imbedded Pragmatic Alzheimer’s Disease and Alzheimer’s Related Dementia (AD/ADRD) Clinical Trials (IMPACT) Collaboratory, in partnership with the Alzheimer’s Association, convened a Lived Experience Panel (LEP), a group of 9‐12 individuals, including people living with cognitive symptoms, proxies representing people with an advanced cognitive disorder or who are deceased, and care partners of a person living with dementia. The aim was for the LEP members to share their experiences with research, inform the development of research priorities, and provide input on conducting embedded pragmatic clinical trials (ePCTs) of dementia care interventions. Given the importance of providing a space for people with lived experiences to share their thoughts and recommendations, we continue to report on the final stage of LEP in its original design.

**Method:**

The recruitment and selection process, as well as the initial convening of the LEP have been reported in the Journal of the American Geriatrics Society. Since that publication, two additional panel series have concluded. In the recent series, the LEP addressed the intersections of dementia care interventions on the topics of health equity and goal‐concordant care. Summary reports of the LEP meetings are available on the IMPACT website. Currently the LEP is convening for a series to assess LEP members’ changes in perspectives about research as a result of participating in the LEP activities. In addition, the current series will gather their recommendations for improvement on the LEP as a model to improve ePCTs.

**Result:**

One summary report was released, titled, “Voices of the Lived Experience Panel: Health Equity in Dementia Care and Research” and one summary report related to goal‐concordant care is in progress. Panelists identified the importance of recognizing diversity of diagnoses within AD/ADRD, as well as discussing opportunities and limitations of evaluating goal‐concordant care for people living with cognitive symptoms and their care partners.

**Conclusion:**

IMPACT’s LEP provides a model for the collaborative engagement of people with lived experiences related to dementia. Results from the LEP reports should be widely shared with the dementia research community to promote further work of including people with lived experiences as research partners.

